# Comparison of Methods for Renal Risk Prediction in Patients with Type 2 Diabetes (ZODIAC-36)

**DOI:** 10.1371/journal.pone.0120477

**Published:** 2015-03-16

**Authors:** Ineke J. Riphagen, Nanne Kleefstra, Iefke Drion, Alaa Alkhalaf, Merel van Diepen, Qi Cao, Klaas H. Groenier, Gijs W. D. Landman, Gerjan Navis, Henk J. G. Bilo, Stephan J. L. Bakker

**Affiliations:** 1 Department of Internal Medicine, Division of Nephrology, University of Groningen, University Medical Center Groningen, Groningen, The Netherlands; 2 Diabetes Centre, Isala Clinics, Zwolle, The Netherlands; 3 Langerhans Medical Research Group, Zwolle, The Netherlands; 4 Department of Internal Medicine, University of Groningen, University Medical Center Groningen, Groningen, The Netherlands; 5 Department of Clinical Epidemiology, Leiden University Medical Center, Leiden, The Netherlands; 6 Department of Epidemiology, University of Groningen, University Medical Center Groningen, Groningen, The Netherlands; 7 Department of General Practice, University of Groningen, University Medical Center Groningen, Groningen, The Netherlands; University of Perugia, ITALY

## Abstract

**Background:**

Patients with diabetes are at high risk of death prior to reaching end-stage renal disease, but most models predicting the risk of kidney disease do not take this competing risk into account. We aimed to compare the performance of Cox regression and competing risk models for prediction of early- and late-stage renal complications in type 2 diabetes.

**Methods:**

Patients with type 2 diabetes participating in the observational ZODIAC study were included. Prediction models for (micro)albuminuria and 50% increase in serum creatinine (SCr) were developed using Cox regression and competing risk analyses. Model performance was assessed by discrimination and calibration.

**Results:**

During a total follow-up period of 10 years, 183 out of 640 patients (28.6%) with normoalbuminuria developed (micro)albuminuria, and 22 patients (3.4%) died without developing (micro)albuminuria (i.e. experienced the competing event). Seventy-nine out of 1,143 patients (6.9%) reached the renal end point of 50% increase in SCr, while 219 (19.2%) died without developing the renal end point. Performance of the Cox and competing risk models predicting (micro)albuminuria was similar and differences in predicted risks were small. However, the Cox model increasingly overestimated the risk of increase in SCr in presence of a substantial number of competing events, while the performance of the competing risk model was quite good.

**Conclusions:**

In this study, we demonstrated that, in case of substantial numbers of competing events, it is important to account for the competing risk of death in renal risk prediction in patients with type 2 diabetes.

## Introduction

Diabetic nephropathy occurs in 20 to 40% of patients with diabetes and is a leading cause of end-stage renal disease (ESRD) [1]. Diabetic nephropathy is characterized by development of proteinuria with a subsequent decline in glomerular filtration rate, which progresses over a long period of time (i.e. 10 to 20 years) [2]. Early identification of patients at risk for diabetic nephropathy may allow optimization of preventive measures to reduce the incidence and progression of diabetic nephropathy. Over the past decades, risk prediction has gained increasing attention and several prediction models have been developed to predict the risk of chronic kidney disease (CKD) [3–6].

Several studies have indicated that patients with diabetes and micro- or macroalbuminuria are at a particularly high risk of death prior to reaching ESRD [7,8]. In the presence of such a substantial competing risk, standard survival predictions may profoundly overestimate the risk of the event of interest (i.e. renal disease) because subjects that die before experiencing the renal event are treated as if they could experience the renal event in the future [9]. To accurately predict absolute risks, it is important to account for the presence of competing risks when performing survival analyses for risk prediction in nephrology [9–11].

However, existing models predicting the risk of kidney disease do not take this potential competing risk of death into account. Therefore, our aim was to compare the predictive performance of basic Cox regression and competing risk models for 10-year risk prediction of (micro)albuminuria, as marker for early-stage renal complications, and 50% increase in serum creatinine (SCr), as marker for late-stage renal complications in a cohort of patients with type 2 diabetes.

## Materials and Methods

### Study group

This study included data from the Zwolle Outpatient Diabetes Project Integrating Available Care (ZODIAC) study. In 1998, the ZODIAC study was initiated in the Zwolle region of the Netherlands. The design and details of this study have been published elsewhere [12]. In this study, general practitioners were assisted by hospital-based diabetes specialist nurses in their care of patients with type 2 diabetes. Patients with a very short life expectancy (including patients with active cancer) or insufficient cognitive abilities were excluded from participation. A total of 1,143 patients with type 2 diabetes that were treated in primary care were included in this prospective cohort study. The ZODIAC study and the informed consent procedure was approved by the local medical ethics committee of the Isala Clinics, Zwolle, the Netherlands. Verbal informed consent was obtained for all patients by the participating diabetes specialist nurses and the consent was documented in the patients records. According to Dutch law, written informed consent was not necessary for this type of study in 1998. All data were analyzed anonymously.

### Data collection and measurements

Baseline data were collected in 1998 and 1999 consisting of a full medical history including macrovascular complications, use of medication, and tobacco consumption as described previously [13]. Patients were considered to have a history of macrovascular complications if they had a history of angina pectoris, myocardial infarction, percutaneous transluminal coronary angioplasty, coronary artery bypass grafting, stroke, or transient ischemic attack. Laboratory and physical assessment data included a non-fasting lipid profile, glycated hemoglobin (HbA_1c_), serum creatinine (SCr), urinary albumin-to-creatinine ratio (ACR), blood pressure, weight, and height. SCr was measured by a kinetic colorimetric Jaffe method (Modular P Analyzer, Roche Almere, the Netherlands), urinary albumin concentration was measured using immunonephelometry (Behring Nephelometer; Mannheim, Germany), and blood pressure was measured twice with a Welch Allyn sphygmomanometer in the supine position after at least 5 minutes of rest. The creatinine-based Chronic Kidney Disease Epidemiology Collaboration (CKD-EPI) equation was used to estimate glomerular filtration rate (eGFR) [14]. To calculate the eGFR, serum creatinine levels were reduced by 5%, because serum creatinine measurements in this study were not standardized to isotope dilution mass spectrometry [15]. In 2009, vital status and cause of death were retrieved from records maintained by the hospital and the general practitioners. Cause of death was coded according to the International Classification of Diseases, 9^th^ revision (ICD-9).

### Clinical end points

In this study, we examined two clinical end points reflecting early- and late-stage renal complications in type 2 diabetes: development of (micro)albuminuria and progressive renal function loss, respectively. ACR and SCr were measured annually during the follow-up period of 10 years. If data on follow-up of ACR and SCr were incomplete, patients were censored at time of last ACR or SCr measurement. (Micro)albuminuria was defined as an albumin-to-creatinine ratio >2.5 mg/mmol for men and >3.5 mg/mmol for women. We considered patients to have developed (micro)albuminuria if 1) patients with normoalbuminuria at baseline had albuminuria in two consecutive follow-up years, 2) patients with normoalbuminuria at baseline developed albuminuria in one single follow-up year, followed by initiation of treatment with an ACEi or ARB in the same year, or 3) patients with normoalbuminuria who received ACEi/ARB treatment at baseline developed albuminuria in one of the follow-up years. Progressive renal function loss was defined as 50% increase of the baseline serum creatinine level in two consecutive follow-up years, which persisted or increased during follow-up. Overlap between the early- and late-stage renal end points was assessed using the χ2 test.

### Statistical analysis

Statistical analyses were performed using SPSS version 19.0 for Windows (SPSS inc., Chicago, Illinois, USA), STATA version 11.0 (StataCorp LP, TX, USA), and R version 3.0.1 (Vienna, Austria) (http://cran.r-project.org/). Results were expressed as mean ± standard deviation or median [interquartile range] for normally and non-normally distributed data, respectively. Nominal data are presented as the total number of patients (percentage).

### Model development

We developed baseline risk scores predicting the 10-year risk of two clinical end points reflecting early- and late-stage renal complications: 1) (micro)albuminuria and 2) 50% increase of baseline SCr. The models predicting the 10-year risk of (micro)albuminuria were developed in 640 patients with normoalbuminuria at baseline. For the models predicting 50% increase in SCr, the whole data set was used. For model development, we used multivariable Cox regression and competing risk analyses using the Fine and Gray method [16]. In multivariable Cox regression analyses, subjects who die before developing the event of interest (i.e. the renal event) are censored at time of death. In competing risk analyses, subjects who experience a competing event (i.e. death) remain in the risk set (instead of being censored) [11,17].

As possible predictors, we used variables that have been suggested in literature to be renal risk factors: age, gender, smoking, body mass index (BMI), macrovascular complications, systolic blood pressure (SBP), use of antihypertensive medication, use of ACEi/ARB, total cholesterol-to-HDL ratio, duration of diabetes, HbA_1c_, eGFR, and ACR [18–21]. Candidate variables were not tested for significance in univariable models before deemed eligible, as this could introduce bias and result in overfitting of the models [22–24]. Continuous variables were entered as continuous predictors in the model development process. Because predictors that are highly correlated with others may contribute little independent information [22], we tested for correlations between candidate variables. Since we found no collinearity (i.e. ρ>0.8) between candidate predictors, none of the selected candidate predictors were excluded beforehand. Since several of the selected candidate predictors contained missing values, multiple imputation (fully conditional specification [MCMC]) was used to obtain 10 imputed datasets [25,26]. Multiple imputation was performed under the assumption that the data are missing at random.

Model development was performed by applying the backward stepwise selection procedure in the multiple imputed dataset as recommended by Wood *et al*. [27]. Rubin’s rules were used to obtain pooled estimates of the regression coefficients and their standard errors across the 10 imputed datasets [27,28]. The backward stepwise selection procedure consists of backward selection, followed by forward selection and iterates if necessary [27]. Variable exclusion in the backward stepwise selection procedure was set to a *P* value of 0.1, the *P* value for subsequent variable inclusion was set to 0.1. Age was retained as a predictor in each model.

As sensitivity analyses we used forward, forward stepwise, and backward selection as additional methods for model derivation. In the forward selection, each candidate predictor is tested for inclusion in the model. The procedure starts with a null model followed by subsequent inclusion of the most significant of the candidate predictors, as long as every new predictor meets the pre-specified significance level, until no remaining variable is significant when added to the model [27]. A drawback of the forward selection procedure is that included predictors may become non-significant after addition of new predictors [27]. In the forward stepwise selection procedure, non-significant variables may be dropped after inclusion of other predictors. The backward procedure starts with a model with all candidate predictors followed by subsequent exclusion of the least significant predictors in the model until all variables retained in the model are significant [27]. The pre-specified significance levels for entry in the forward selection procedure and for variable exclusion in the backward selection procedure were set to 0.1.

### Model performance

Model performance was assessed by discrimination and calibration. Discrimination, a measure to evaluate how well a model distinguishes between patients with and without the outcome, was assessed using Harrell’s C-statistic [9,29]. The interpretation of the Harrell’s C-statistic is similar to that of the area under the curve (AUC) of the receiver operator curve (ROC). A value of 1.0 indicates perfect prediction and a value of 0.5 indicates that patients are correctly classified in 50% (i.e. as good as chance). The adapted C-statistic of Wolbers *et al*. [9] was used for the competing risks models. This adapted C-statistic accounts for the fact that competing events prevent the occurrence of the event of interest, whereas the traditional Harrell’s C-statistic could falsely suggest better predictive ability of the event of interest in case of strong competing risks [9]. Calibration, a measure to evaluate how well predicted probabilities agree with observed risks, was determined by comparing the mean predicted survival with the mean observed risk by deciles of predicted risk. Observed risks for renal complications were calculated using the cumulative incidence function, which accounts for the competing risk of death [9,11]. Finally, we compared absolute predicted risks as obtained by the Cox regression and competing risk models.

## Results

### Patient characteristics

Baseline patient characteristics for the subgroup with normoalbuminuria and the total study population are presented in Table 1. Patient characteristics of the subgroup with normoalbuminuria and the total study population are similar. In the total study population, only 22.7% of the subjects used an ACEi or ARB at baseline, and 10.9% used a lipid lowering agent at baseline. The count and percentage of missing values in the selected candidate predictors are presented in S1 Table.

**Table 1:** Baseline patient characteristics of the ZODIAC study population.

	Normoalbuminuria		All patients	
	(n = 640)		(n = 1,143)	
	Mean ± SD*	Range	Mean ± SD*	Range
**Demographics**				
Age (years)	66 ± 12	21–97	68 ± 12	21–97
Male gender (n, %)	252 (39.4)	-	489 (42.8)	-
**Body composition**				
BMI (kg/m^2^)	29.0 ± 4.6	17.7–46.7	28.9 ± 4.8	16.2–47.2
**Blood pressure**				
Systolic blood pressure (mmHg)	150 ± 24	100–230	155 ± 25	95–240
Diastolic blood pressure (mmHg)	84 ± 10	50–120	84 ± 10	50–120
Use of ACEi or ARB (n, %)	128 (20.0)	-	260 (22.7)	-
Use of anti-hypertensive drugs (n, %)	269 (42.0)	-	552 (48.3)	-
**Glucose homeostasis**				
Duration of diabetes (years)	5 [3–10]	0–51	6 [3–11]	0–58
HbA_1c_ (mmol/mol)	55 [48–65]	29–113	56 [49–67]	29–120
HbA_1c_ (%)	7.2 [6.5–8.1]	4.8–12.5	7.3 [6.6–8.3]	4.8–13.1
**Lipids**				
Total cholesterol (mmol/L)	5.7 ± 1.1	2.7–11.8	5.7 ± 1.1	2.7–11.8
HDL cholesterol (mmol/L)	1.2 ± 0.5	0.5–8.9	1.2 ± 0.4	0.5–8.9
Triglycerides (mmol/L)	2.1 [1.5–3.0]	0.5–15.9	2.2 [1.5–3.1]	0.5–15.9
Cholesterol-HDL ratio	5.1 ± 1.5	1.3–11.2	5.2 ± 1.6	1.3–13.6
Use of lipid lowering drugs (n, %)	68 (10.6)	-	125 (10.9)	-
**Renal function**				
Serum creatinine (μmol/L)	93 ± 18	59–228	97 ± 23	56–293
eGFR (mL/min/1.73m^2^)	68 ± 17	23–120	67 ± 17	16–120
ACR (mg/mmol)	1.2 [0.7–1.8]	0–3.4	2.2 [1.0–7.3]	0–588
Albuminuria (n, %)	-	-	457 (40.0)	-
**Other**				
Smoking (n, %)	122 (19.1)	-	211 (18.5)	-
Macrovascular complications (n, %)	192 (30.0)	-	401 (35.1)	-

### Clinical end points

After a total follow-up period of 10 years, 183 out of the 640 patients (28.6%) with normoalbuminuria at baseline developed (micro)albuminuria. A total of 22 patients (3.4%) died without developing (micro)albuminuria (i.e. experienced the competing event of death). A total of 78 patients (12.2%) had incomplete ACR follow-up data and were censored at time of last ACR measurement. Because several patients experienced a renal event or were censored due to incomplete follow-up before the end of the total follow-up period, the median follow-up time was slightly lower (i.e. 8.8 [IQR 4.3–9.9] years) than the total follow-up period of 10 years.

After a total of 10 years of follow-up (median 8.9 [3.7–9.9] years), 79 out of 1,143 patients (6.9%) reached the renal end point of 50% increase in baseline serum creatinine. A total of 219 patients (19.2%) died without developing late-stage renal complications. A total of 112 patients (9.8%) had incomplete SCr follow-up data and were censored at time of the last SCr measurement. Forty-eight patients that developed 50% increase in SCr had (micro)albuminuria at baseline and 10 patients developed (micro)albuminuria prior to developing 50% increase in SCr. Thus, a total of 58 out of 79 patients (73.4%) developed (micro)albuminuria prior to developing 50% increase in SCr, whereas 18 out of 79 patients (22.8%) did not develop (micro)albuminuria prior to developing 50% increase in SCr (*P* = 0.003).

### Model development

The final models predicting early- and late-stage renal complications in patients with type 2 diabetes developed using backward stepwise selection are shown in Table 2 (see also S1 Item). The models predicting early-stage renal complications (i.e. [micro]albuminuria) included the predictors age, gender, smoking, macrovascular complications, SBP, HbA_1c_, and ACR (Table 2). The models predicting late-stage renal complications included five predictors: age, macrovascular complications, BMI, SBP, and ACR (Table 2).

**Table 2:** Developed risk prediction models for early-stage renal complications ([micro]albuminuria) and late-stage renal complications (50% increase in baseline serum creatinine) in patients with type 2 diabetes.

	(Micro)albuminuria						50% increase in baseline SCr					
	(n_events_/n_deaths_/n_total_ = 183/22/640)						(n_events_/n_deaths_/n_total_ = 79/219/1,143)					
	Cox Regression			Competing Risk			Cox Regression			Competing Risk		
Risk factors	β	HR (95% CI)	P	β	SHR (95% CI)	P	β	HR (95% CI)	P	β	SHR (95% CI)	P
Age (per 10 years)	0.42	1.52 (1.28–1.79)	<0.001	0.35	1.42 (1.20–1.68)	<0.001	0.37	1.45 (1.11–1.88)	0.006	0.16	1.18 (0.94–1.48)	0.15
Gender (male vs female)	0.49	1.63 (1.17–2.27)	0.004	0.46	1.59 (1.15–2.21)	0.006	-	-	-	-	-	-
BMI (per kg/m^2^)	-	-	-	-	-	-	0.07	1.07 (1.02–1.12)	0.005	0.06	1.07 (1.02–1.12)	0.005
SBP (per 10 mmHg)	0.15	1.16 (1.10–1.24)	<0.001	0.15	1.17 (1.10–1.24)	<0.001	0.11	1.12 (1.02–1.23)	0.02	0.11	1.12 (1.03–1.21)	0.01
HbA_1c_ (per 10 mmol/mol)*	0.13	1.14 (1.02–1.28)	0.03	0.10	1.11 (0.99–1.25)	0.09	-	-	-	-	-	-
ACR (per log_10_ mg/mmol)	0.88	2.41 (1.26–4.62)	0.008	0.86	2.36 (1.84–3.03)	0.02	0.92	2.50 (1.79–3.50)	<0.001	0.75	2.12 (1.47–3.06)	<0.001
Smoking (yes vs no)	0.45	1.57 (1.07–2.30)	0.02	0.47	1.61 (1.10–2.34)	0.01	-	-	-	-	-	-
MVC (yes vs no)	0.42	1.52 (1.11–2.09)	0.009	0.42	1.53 (1.11–2.11)	0.01	0.66	1.94 (1.22–3.08)	0.005	0.46	1.59 (0.98–2.58)	0.06

As sensitivity analyses, forward, forward stepwise, and backward selection were used as additional methods for model derivation. The Cox regression model predicting (micro)albuminuria developed using forward selection was not different from the model developed using the backward stepwise selection procedure. The competing risk model predicting (micro)albuminuria developed using forward selection did, however, not contain HbA_1c_ as predictor, and the models predicting 50% increase in SCr developed using forward regression contained total cholesterol-to-HDL ratio as additional predictor, but this predictor lost significance (*P* value>0.1) after addition of BMI to the model. The Cox regression and competing risk models developed using forward stepwise selection and backward selection were not different from the models developed using the backward stepwise selection procedure.

### Model performance

The discriminative performance of the models was assessed using the Harrell’s C statistic. The Harrell’s C-statistics for both the final Cox regression and competing risk model predicting (micro)albuminuria were 0.69 (0.65–0.72). A Harrell’s C-statistic of 0.69 indicates that 69% of the subjects are correctly classified using the prediction models (i.e. moderate to good discrimination). The Harrell’s C-statistics for the Cox regression model predicting increase in SCr was 0.73 (0.68–0.78) and the C-statistic for the competing risk model was 0.74 (0.69–0.79), indicating that 73% and 74% of the patients are correctly classified (i.e. good discrimination).

**Fig 1:**
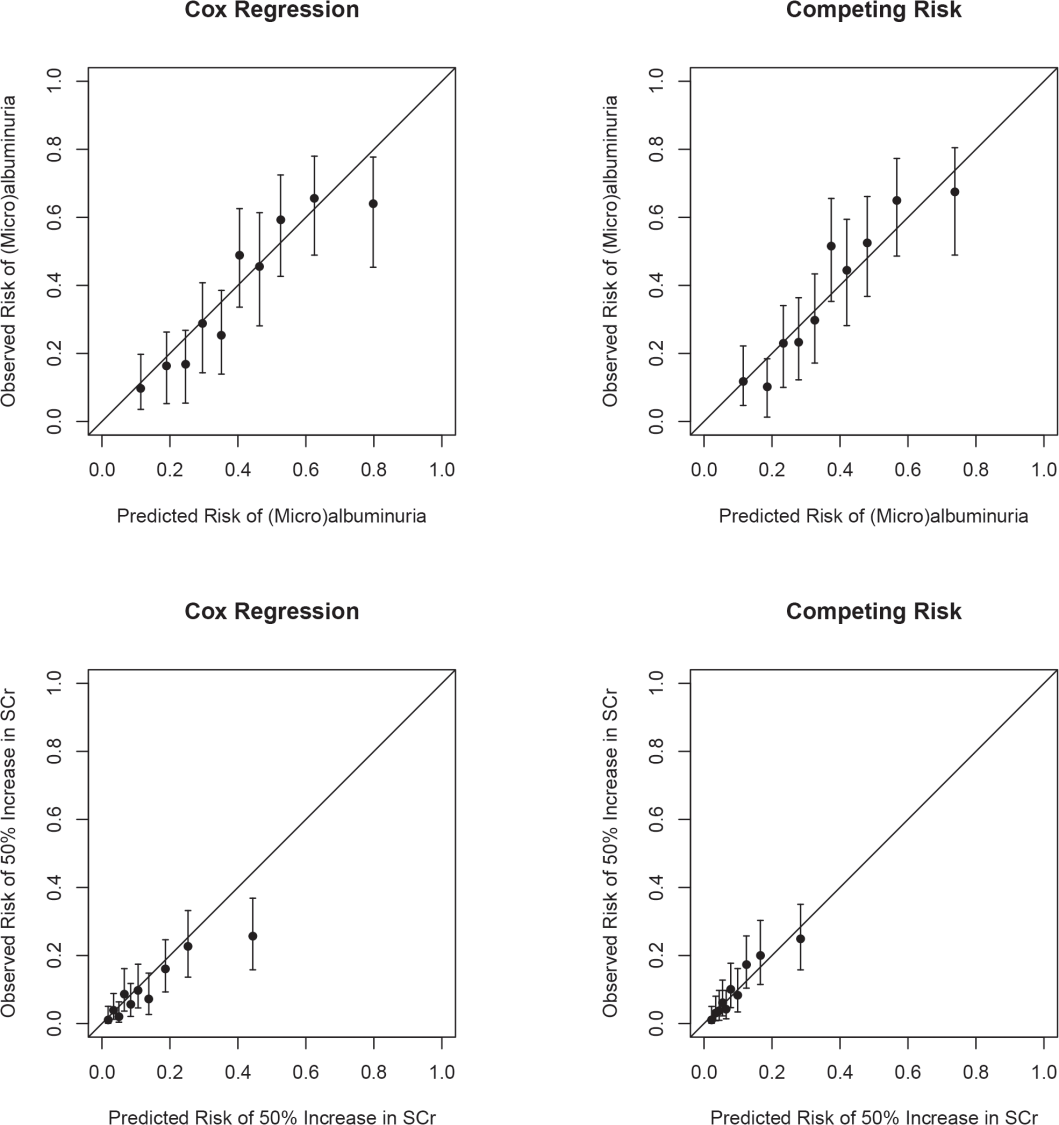
Calibration plots of mean predicted risk versus mean observed risk (cumulative incidence) and corresponding 95% confidence intervals presented according to deciles of predicted risk for the models predicting (micro)albuminuria and 50% increase in serum creatinine (SCr).

Calibration was determined by comparing mean predicted 10-year risk with mean observed 10-year risk by deciles of predicted risk. The calibration plots for the developed renal risk scores are shown in Fig. 1. The calibration plots of the Cox regression model predicting (micro)albuminuria indicates moderate calibration, with most of the predicted risk estimates within the 95% CI’s of the corresponding observed risk estimates, while the predicted risks of the competing risk model are well within the 95% CI’s of the observed risk estimates indicating good calibration. The Cox regression model seemed to increasingly overestimate the risk of 50% increase in SCr in the presence of a substantial number of competing events, while the predicted risks of the competing risk model are well within the 95% CI’s of the observed risk estimates.

**Fig 2:**
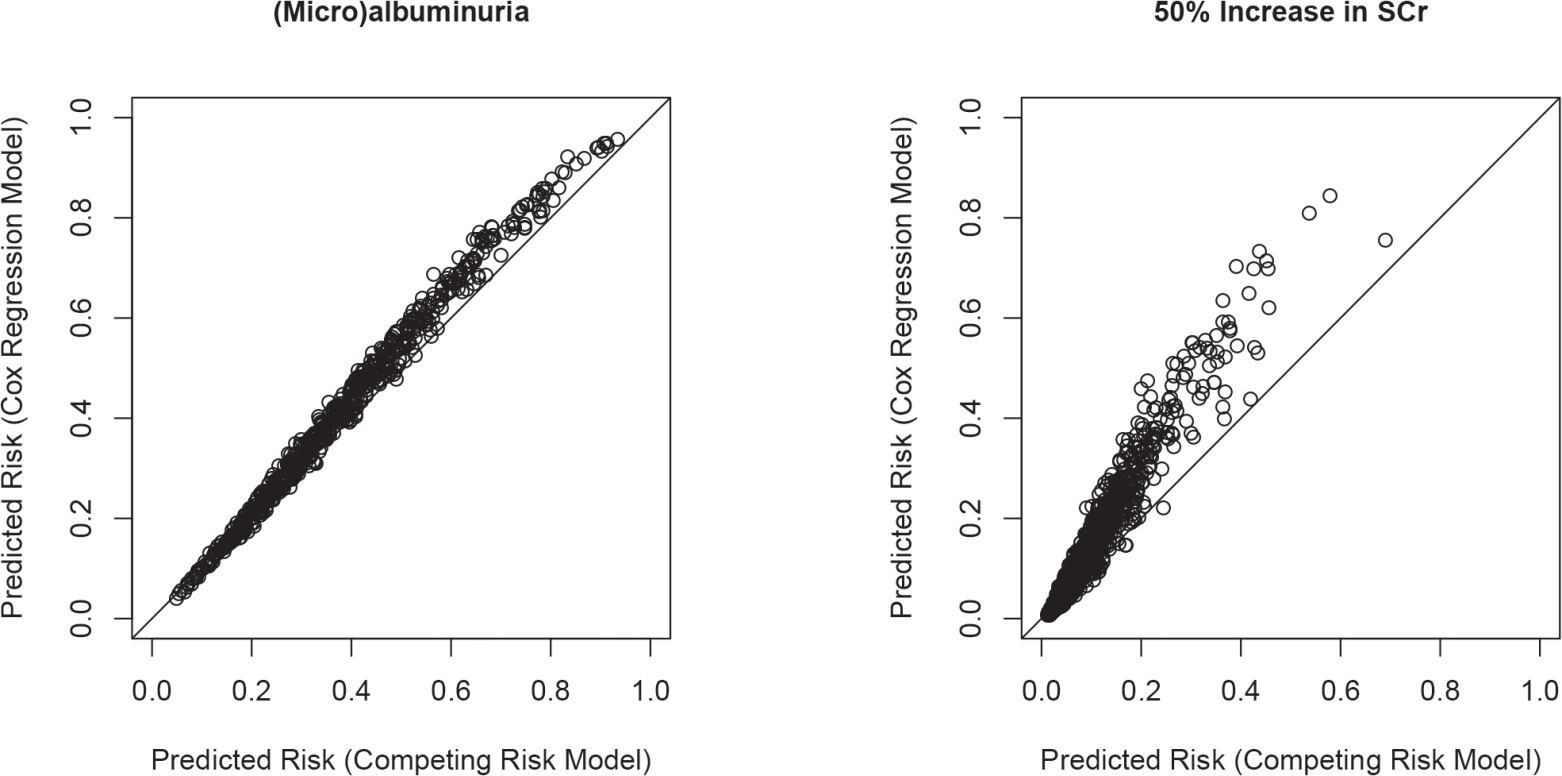
Scatter plots of predicted risks (competing risk models versus Cox regression models) for early stage (i.e. [micro]albuminuria) and late stage renal complications (i.e. 50% increase in SCr).

Furthermore, we compared absolute predicted risks as obtained by the Cox regression and competing risk models (Fig. 2). The differences in absolute predicted risks as obtained by the Cox regression and competing risk models for (micro)albuminuria were small (Fig. 2), with differences in absolute predicted risks ranging from −2.0% to 12.3%. The differences in absolute predicted risks as obtained by the Cox regression and competing risk models for late-stage renal complications were more pronounced (Fig. 2), with differences in absolute predicted risks ranging from −2.9% to 31.2%.

## Discussion

In this study, we compared the predictive performance of Cox regression and competing risk models for 10-year risk prediction of early- and late-stage renal complications in patients with type 2 diabetes. In the presence of a limited number of competing events, as in risk prediction of early-stage renal complications (i.e. [micro]albuminuria), the performance of the Cox regression and competing risk models was similar and the differences in absolute predicted risks were small. However, in the presence of a substantial number of competing events, as in risk prediction of the late-stage renal end point of 50% increase in SCr, the Cox regression model increasingly overestimated the absolute predicted risks of the renal end point, while the competing risk model did not suffer from risk overestimation. This indicates that, in case of substantial numbers of competing events, it is important to account for the competing risk of death when performing survival analyses for renal risk prediction in patients with type 2 diabetes.

Over the past decades, risk prediction has gained increasing attention and several prediction models have been developed to predict CKD in the general population [3–5]. One study based on data from the ADVANCE cohort developed risk prediction models for 5-year risk prediction of new-onset albuminuria and major kidney-related outcomes in patients with type 2 diabetes [6]. Keane et al. developed risk scores for ESRD and the combined end point of ESRD and death in patients with type 2 diabetes and nephropathy based on data of the RENAAL study [30]. The models developed in the ADVANCE and RENAAL cohorts both included eGFR as predictor. In our study population, eGFR lost significance after inclusion of age in the prediction models, and was therefore not included in our final models. In contrast to our study, age was not included as a predictor in the final ADVANCE and RENAAL prediction models. As eGFR declines with increasing age, inclusion of age in the prediction models explains a large part of the predictive abilities of eGFR. This likely explains the discrepancy concerning inclusion of age or eGFR in our risk prediction models versus the ADVANCE and RENAAL models.

Several studies have indicated that it is important to be aware of the potential presence of competing risks when performing survival analyses for risk prediction in nephrology [10,11]. It has been demonstrated that patients with diabetes and micro- or macroalbuminuria are at a particularly high risk of death prior to reaching ESRD [7,8]. In the presence of a competing event (i.e. death before reaching the renal event), the risk of renal complications could be overestimated when standard survival analyses are used. In the current study, we compared the predictive performance of standard Cox regression and competing risk models that account for the potential competing risk of death. The performance of the Cox regression and competing risk models predicting early-stage renal complications (i.e. [micro]albuminuria) was similar and the difference in absolute predicted risks was small. In contrast, there was a substantial number of patients that died without developing late-stage renal complications (i.e. competing events). Consequently, the differences in absolute predicted risks, as obtained by the Cox regression and competing risk models, were more pronounced. The absolute predicted risks for late-stage renal complications were overestimated when the standard Cox regression model was used for renal risk prediction. For the ADVANCE risk score, the authors primarily used the standard Cox regression method for model development [6]. The Fine and Gray method was used for sensitivity analyses, for which it was reported that subhazard ratios for these analyses were similar to hazard ratios and that absolute predicted risks were unchanged after taking the competing risk of death into account [6]. The difference in follow-up time between our study and the ADVANCE study (10 versus 5 years, respectively) and the number of patients that died without developing the late-stage renal complications (19.2% in ZODIAC versus 8.7% in ADVANCE) are possible explanations for the differences in results.

The present study has several limitations. Our study population, consisting of 1,143 patients with type 2 diabetes, was relatively small. As a consequence of the small population sample, and the long time interval before development of diabetic nephropathy [2,7], the number of patients that reached doubling of SCr or ESRD was limited in this study population. To identify patients with the most progressive renal function loss within a period of 10 years, we used a surrogate end point of 50% increase of baseline SCr. Because of the relatively small study population, we compared several model development strategies. The models developed using forward selection contained an additional predictor that lost significance after addition of BMI to the model. Inclusion of variables that may become non-significant after addition of new variables is a well-known drawback of the forward selection procedure [27]. Furthermore, as a consequence of the small study population, we were not able to assess the discriminative performance in subgroups of patients. Finally, we acknowledge that external validation of prediction models is important and necessary before implementation of prediction models in guidelines or clinical practice. External validation of the developed models could demonstrate how the models perform in external datasets. Strengths of the present study are the relatively long follow-up period of 10 years, which corresponds with the long period of time over which diabetic nephropathy progresses, and the use of both Cox regression and competing risk analyses that accounts for the potential competing risk of death.

In conclusion, we compared the predictive performance of standard Cox regression and competing risk models predicting the 10-year risk of early-stage (i.e. [micro]albuminuria) and late-stage (i.e. 50% increase in serum creatinine) renal complications in patients with type 2 diabetes treated in primary care. In the presence of a limited number of competing events, we found no essential differences between the Cox regression and competing risk models predicting the risk of early-stage renal complications in type 2 diabetes. However, in the presence of a substantial number of competing events, the use of standard Cox regression analyses for risk prediction of late-stage renal complications resulted in overestimated predicted risks. The results of this study indicate that, in case of substantial numbers of competing events, it is important to account for the competing risk of death when performing survival analyses for renal risk prediction in patients with type 2 diabetes.

## Supporting Information

S1 Item Final risk prediction models for 10-year risk prediction of early-stage renal complications (i.e. [micro]albuminuria) and late-stage renal complications (i.e. 50% increase in serum creatinine) in type 2 diabetes. (DOCX)

S1 Table Missing values of selected candidate predictors for model development of early-stage renal complications (i.e. [micro]albuminuria) and late-stage renal complications (i.e. 50% increase in serum creatinine) in the ZODIAC study. (DOCX)
