# Supplementary material for: Comparison of Methods for Renal Risk Prediction in Patients with Type 2 Diabetes (ZODIAC-36)
Source: PLoS One. 2015 Mar 16;10(3):e0120477. doi: 10.1371/journal.pone.0120477 (PMC4361549; doi:10.1371/journal.pone.0120477)
Supplement: S1 Item — (DOCX) [file pone.0120477.s001.docx]

**Supporting Information S1 Item.** Final risk prediction models for 10-year risk prediction of early-stage renal complications (i.e. [micro]albuminuria) and late-stage renal complications (i.e. 50% increase in serum creatinine) in type 2 diabetes.

**(Micro)albuminuria**

**Cox Regression**

P_microalbuminuria_ = 1 – exp(-0.0017 * exp(Xb_COX1_))

Xb_COX1_ = 0.0415 * age (years) + 0.489 (if male) + 0.0152 * systolic blood pressure (mmHg) + 0.0130 * HbA_1c_ (mmol/mol) + 0.880 * log_10_ ACR (mg/mmol) + 0.450 (if smoker) + 0.419 (if history of macrovascular complications) – 5.660.

**Competing Risk**

P_microalbuminuria_ = 1 – exp(-0.0021 * exp(Xb_CR1_))

Xb_CR1_ = 0.0350 * age (years) + 0.465 (if male) + 0.0153 * systolic blood pressure (mmHg) + 0.0103 * HbA_1c_ (mmol/mol) + 0.859 * log_10_ ACR (mg/mmol) + 0.473 (if smoker) + 0.424 (if history of macrovascular complications) – 5.220.

**50% increase in serum creatinine**

**Cox Regression**

P_50%increaseSCr_ = 1 – exp(-0.0001 * exp(Xb_COX2_))

Xb_COX2_ = 0.0370 * age (years) + 0.0654 * BMI (kg/m^2^) + 0.0110 * systolic blood pressure (mmHg) + 0.918 * log_10_ ACR (mg/mmol) + 0.661 (if history of macrovascular complications) – 6.782.

**Competing Risk**

P_50%increaseSCr_ = 1 – exp(-0.0015 * exp(Xb_CR2_))

Xb_CR2_ = 0.0165 * age (years) + 0.0642 * BMI (kg/m^2^) + 0.0110 * systolic blood pressure (mmHg) + 0.753 * log_10_ ACR (mg/mmol) + 0.462 (if history of macrovascular complications) – 5.211.
