# Supplementary material for: Comparison of Methods for Renal Risk Prediction in Patients with Type 2 Diabetes (ZODIAC-36)
Source: PLoS One. 2015 Mar 16;10(3):e0120477. doi: 10.1371/journal.pone.0120477 (PMC4361549; doi:10.1371/journal.pone.0120477)
Supplement: S1 Table — (DOCX) [file pone.0120477.s002.docx]

**Supporting Information S1 Table.** Missing values of selected candidate predictors for model development of early-stage renal complications (i.e. [micro]albuminuria) and late-stage renal complications (i.e. 50% increase in serum creatinine) in the ZODIAC study.

| **Candidate predictor** | **Normoalbuminuria**  **(N = 640)** |  | **All patients**  **(N = 1,143)** | |
| --- | --- | --- | --- | --- |
|  | **N (%)** |  | | **N (%)** |
| **Demographics** |  |  | |  |
| Age (years) | 0 (0) |  | | 0 (0) |
| Gender | 0 (0) |  | | 0 (0) |
| **Body composition** |  |  | |  |
| BMI (kg/m^2^) | 1 (0.2) |  | | 3 (0.3) |
| **Blood pressure** |  |  | |  |
| Systolic blood pressure (mmHg) | 2 (0.3) |  | | 2 (0.2) |
| Use of ACEi/ARB | 4 (0.6) |  | | 5 (0.4) |
| **Glucose homeostasis** |  |  | |  |
| HbA_1c_ (mmol/mol) | 0 (0) |  | | 3 (0.3) |
| Diabetes duration (years) | 1 (0.2) |  | | 8 (0.7) |
| **Lipids** |  |  | |  |
| Cholesterol-HDL ratio | 0 (0) |  | | 4 (0.3) |
| **Renal function** |  |  | |  |
| SCr (µmol/L) | 0 (0) |  | | 2 (0.2) |
| eGFR (mL/min/1.73m^2^) | 0 (0) |  | | 2 (0.2) |
| ACR (mg/mmol) | 0 (0) |  | | 46 (4.0) |
| **Other** |  |  | |  |
| Smoking | 13 (2.0) |  | | 19 (1.7) |
| Macrovascular complications | 0 (0) |  | | 0 (0) |
| **Outcome** |  |  | |  |
| (Micro)albuminuria | 89 (13.9) |  | | NA |
| 50% increase in SCr | NA |  | | 13 (1.1) |
| Abbreviations: ACEi, angiotensin-converting enzyme inhibitor; ACR, urinary albumin-to-creatinine ratio; ARB, angiotensin receptor blocker; BMI, body mass index; eGFR, estimated glomerular filtration rate; HDL, high-density lipoprotein; NA, not applicable; SCr, serum creatinine. | | | | |
